# Predictors of Antenatal Care, Skilled Birth Attendance, and Postnatal Care Utilization among the Remote and Poorest Rural Communities of Zambia: A Multilevel Analysis

**DOI:** 10.3389/fpubh.2017.00011

**Published:** 2017-02-09

**Authors:** Choolwe Jacobs, Mosa Moshabela, Sitali Maswenyeho, Nildah Lambo, Charles Michelo

**Affiliations:** ^1^School of Nursing and Public Health, University of KwaZulu-Natal, Durban, South Africa; ^2^Department of Public Health, School of Medicine, University of Zambia, Lusaka, Zambia; ^3^Africa Centre for Population Health, Mtubatuba, South Africa; ^4^UNICEF, Lusaka, Zambia

**Keywords:** antenatal care, focused antenatal care, skilled birth attendance, postnatal care, maternal health-care utilization

## Abstract

**Objective:**

Optimal utilization of maternal health-care services is associated with reduction of mortality and morbidity for both mothers and their neonates. However, deficiencies and disparity in the use of key maternal health services within most developing countries still persist. We examined patterns and predictors associated with the utilization of specific indicators for maternal health services among mothers living in the poorest and remote district populations of Zambia.

**Methods:**

A cross-sectional baseline household survey was conducted in May 2012. A total of 551 mothers with children between the ages 0 and 5 months were sampled from 29 catchment areas in four rural and remote districts of Zambia using the lot quality assurance sampling method. Using multilevel modeling, we accounted for individual- and community-level factors associated with utilization of maternal health-care services, with a focus on antenatal care (ANC), skilled birth attendance (SBA), and postnatal care (PNC).

**Results:**

Utilization rates of focused ANC, SBA, and PNC within 48 h were 30, 37, and 28%, respectively. The mother’s ability to take an HIV test and receiving test results and uptake of intermittent preventive treatment for malaria were positive predictors of focused ANC. Receiving ANC at least once from skilled personnel was a significant predictor of SBA and PNC within 48 h after delivery. Women who live in centralized rural areas were more likely to use SBA than those living in remote rural areas.

**Conclusion:**

Utilization of maternal health services by mothers living among the remote and poor marginalized populations of Zambia is much lower than the national averages. Finding that women that receive ANC once from a skilled attendant among the remote and poorest populations are more likely to have a SBA and PNC, suggests the importance of contact with a skilled health worker even if it is just once, in influencing use of services. Therefore, it appears that in order for women in these marginalized communities to benefit from SBA and PNC, it is important for them to have at least one ANC provided by a skilled personnel, rather than non-skilled health-care providers.

## Introduction

Poor utilization of maternal health services is frequently associated with maternal deaths and morbidity (1), and yet, most maternal deaths could be prevented if all women successfully utilized maternal health-care services (2). Literature also shows that utilization of maternal and neonatal health (MNH) services can be successfully traced through key services such as attendance of antenatal care (ANC), birth by a skilled health worker, and postnatal care (PNC), all of which are evidently associated with reduction in mortality and morbidity for mothers and their neonates (2–4). According to the World Health Organization’s (WHO) recommendation, every pregnant woman should receive a minimum of four ANC visits in low-risk pregnancies, also known as focused ANC. Focused ANC may be particularly advantageous in resource-limited and marginalized communities, as it requires a minimum of four visits, which are more practical than the traditional ANC (5).

Skilled birth attendance (SBA) is defined as the process by which a woman is provided with adequate care during labor, delivery, and the early postpartum period by a skilled health worker such as a doctor, nurse, or midwife (6). SBA is important for the early identification of signs of complications in order to institute timely management, including referrals to higher levels of care where necessary. In addition, utilization of PNC within 48 h of delivery is known to improve health outcomes for both the mother and the neonate by reducing morbidity and mortality (7). However, despite these essential services being available in health facilities and being made affordable in most developing countries, utilization of ANC, SBA, and PNC remain limited, particularly in developing countries (8). For instance, less than 30% of women are said to access and use PNC services in developing countries (9). The rural populations are the most affected (10). In Zambia, like many other developing countries, utilization of maternal health in rural areas is a challenge. According to the Zambia Demographic Health Survey (ZDHS), only 55% of women attend the WHO-recommended focused ANC visits, 56% are assisted by a skilled birth attendant, and only 54% attend PNC visits (11).

Studies to identify and understand reasons for poor utilization of maternal health-care services among rural populations in developing countries, indicate that patterns of utilizing indicators of maternal health services (ANC, SBA, and PNC) differ across socio demographic, socioeconomic, and cultural context (10, 12–16), including accessibility of these services (2, 17, 18). Factors that influence utilization of maternal health services in rural areas have also been shown to operate at various levels; individual, household, and community (10, 12, 13). According to Jat et al. (12), both individual- and community-level characteristics can cause disparities in the patterns of maternal health-care utilization among rural populations. However, there is lack of evidence on the utilization patterns and predictors of maternal health services among the remote and poorest populations of Zambia. The remote and poorest populations are the most vulnerable and marginalized communities of Zambia. The classification of poorest is based on the analysis of national data on the population distribution of vulnerability, poverty, and deprivation (19). The poorest inhabit households that live on less than a dollar per day (20). Remoteness is determined on the basis of distance from cities and towns, as well as geographical disadvantages, such as being hard to reach, poor road network, poor transportation access, long travel time, and resource-limitations to amenable social services.

Although several studies have been conducted to identify and understand reasons for poor utilization of maternal health-care services in the rural areas (1, 2, 12, 17, 21, 22), studies trying to examine predictors of utilization of the three indicators of pregnancy-related care—antenatal, delivery, and postnatal services—among the remote and poorest rural populations in Zambia in particular are very scarce.

In addition, although, we know that in Zambia, utilization of maternal health services in rural areas is low (11), we do not know the patterns and drivers of utilization of maternal health services in the most remote and poorest rural populations.

Furthermore, we do not know if there are possible existing disparities in the use of the key maternal health-care indicators among these populations. Thus, identifying utilization patterns and the associated factors for the specific indicators of maternal health services is crucial in informing policy and implementation. Therefore, the objective of this study was to examine patterns and predictors of utilization of the three indicators of maternal health-care services among mothers living in the poorest rural and remote populations of Zambia.

## Materials and Methods

### Study Setting

The study was conducted in four rural districts namely Mungwi, Luwingu Samfya, and Chiengi, located in Luapula and the Northern provinces of Zambia. These districts are classified among the 13 poorest in Zambia, where the most vulnerable and marginalized people live. The classification is based on the national distribution of vulnerability, poverty, and deprivation (20). The sampled districts have the highest maternal and child mortality rates (11). The combined estimated population size of these four districts is 580,090 (20). Most of the people in these four districts are peasant farmers, engaged in crop, livestock production, and fishing.

### Study Design and Sampling

The study was a cross-sectional household survey based on the lot quality assurance sampling (LQAS) method. LQAS was used as guided by the WHO and other related studies (23). Using the LQAS method, a district is considered an independent site, and sub-divided into community clusters regarded as supervisory areas. A supervisory area or community cluster constitutes a catchment area with a dedicated health facility responsible for delivering health services. In this study, the four selected districts had a total of 29 community clusters, 9 from both Chiengi and Samfya, 6 from Luwingu, and 5 from Mungwi. Using the WHO LQAS guide, a list of all the villages in each community cluster was retrieved from the 2010 population census, and the sampling proportionate to size technique was followed to randomly select 19 households from each community cluster (23). A sampling frame was used to select households from which the individual samples were taken. The main criterion for inclusion of households was the presence of mothers with children aged 0–5 months and mothers that lived in the study site during pregnancy and delivered their baby within the same study area. In instances where two or more respondents were found in one household, and met the criteria, random sampling was done to select one respondent.

### Data Collection

A pretested structured questionnaire, developed in a local language, was used in face-to-face interviews to collect data from eligible mothers of infants aged 0–5 months. Trained research assistants conducted the interviews, asking questions on utilization of antenatal, delivery, and PNC services, which were provided at no cost in all government health facilities. Collected data included use of focused ANC during pregnancy, SBA at deliveries conducted at home or in a health facility by trained health personnel, and PNC within 48 h after delivery by an appropriate provider. ANC, SBA, and PNC were handled as outcome variables in this study.

The individual explanatory variables included were mother’s age, attendance of school, level of education, literacy level, marital status, HIV test, HIV test results, and intermittent preventive treatment (IPTp). Like many other countries, Zambia adopted a provider-initiated HIV testing and counseling model. On first contact (preferably during first trimester) at ANC clinic, pregnant women are informed about HIV testing during group pretest counseling. Unless a woman “opts out,” providers then perform a rapid HIV test that produces results within 1 h. This service is offered as part of the standard package of ANC (24). In addition, as a preventive measure, it is recommended that pregnant women take three doses of IPTP during pregnancy; with the first dose taken after the first trimester and at least 1 month apart. The choice of explanatory variables is based on previous studies that identified these variables as some of the factors that influence the outcome variable. Therefore, we sought to test the influence of these variables in the context of the study.

Using statistics from the 2010 population census and housing survey, we extracted characteristics of the place of residence and the total population in each community cluster. Place of residence was categorized as central rural or remote rural. For this study, a central rural area is defined as a central administrative area of the district and a remote geographic area as an area further away from the central administrative area of the district. The other variable used at community level was the ratio of primary health centers (PHC) to population, categorized as less than 10,000 or above as per standards of the Ministry of Health in Zambia (25).

### Data Analysis and Modeling

We calculated weighted proportions, to adjust for disproportionate sampling of mothers’ use of the three indicators of maternal health services for each category in the explanatory variables. Univariate analysis was conducted for ANC, SBA, and PNC, taking the weighted odds ratio as the measure of association with the corresponding 95% confidence intervals (95% CI). Since our data had a hierarchical structure, where 551 individual women were located within 29 clustered communities, multilevel modeling was applied in the analysis in order to account for the multistage cluster effects of the LQAS method (12, 26, 27). The two-level model was fitted to assess the influence of measured individual and community cluster factors for each of the three indicators of maternal health services. We estimated the community-level random effects using the *xtmelogit* command in Stata version 13 (Stata Corp. Inc., TX, USA). In the first empty model, no independent variables were included in order to show the total variance in the use of the three maternal health services between the community clusters. In the second full model, fixed effects at individual level were included together with the community cluster-level random effects. The results of fixed effects are presented as odds ratios (ORs) with *P*-values and 95% CIs, and random effects as variance partition coefficients (VPC) (12). The VPC was calculated as:

$${\text{VPC}}_{C}=\frac{\sigma_{c}^{2}}{\sigma_{c}^{2}+3.29}$$

where $\sigma_{c}^{2}$ represents community cluster-level variance.

### Ethical Clearance

Ethical approval was obtained from research ethics committees at the Tropical Disease Research Centre (TDRC; Ref No: TRC/C4/07/2015) in Zambia and the University of KwaZulu-Natal (Ref No: BE363/15) in South Africa. Formal permission was also obtained from the Ministry of Community Development, Mother and Child, and from the traditional leaders through the District Medical Officers of the respective selected districts. In addition, informed consent was obtained from all participants.

## Results

### Characteristics of Participants

A total of 551 women were interviewed. As shown in Table 1, the median age for the participants was 26 years, with a range of 15–54 years. One in five mothers (20%) had never been to school, and mothers, who could not read at all, were as high as 63% among the respondents. Among the mothers, 78% reported to have had an HIV test done during their last pregnancy, but 27% did not receive their HIV test results.

**Table 1:** **Characteristics of participants in relation to utilization of maternal health-care services among the poorest populations of Zambia**.

Variables	Weighted% (*N*)	95% CI
**Age**		
15–24	41 (218)	(36–46)
25–40	53 (287)	(48–58)
41–54	5 (27)	(2–9)
**Marital status**		
Not married	35 (211)	(32–38)
Married	65 (340)	(62–68)
**Ever attended school**		
No	19 (110)	(16–23)
Yes	81 (438)	(77–84)
**Literacy**		
Cannot read at all	63 (252)	(58–68)
Only parts	10 (52)	(7–13)
Whole sentence	27 (146)	(23–32)
**At least one antenatal care (ANC) visit by skilled attendant**		
No	31 (195)	(27–35)
Yes	69 (356)	(65–73)
**At least four ANC visits**		
No	71 (386)	(66–75)
Yes	29 (165)	(25–34)
**Skilled birth attendant**		
No	60 (342)	(56–66)
Yes	39 (209)	(34–44)
**Four ANC visits with at least one skilled attendance**		
No	71 (389)	(67–76)
Yes	29 (162)	(24–33)
**Did an HIV test**		
No	23 (124)	(19–27)
Yes	78 (427)	(73–81)
**Received HIV test results**		
No	27 (154)	(23–32)
Yes	73 (397)	(24–32)
**Received IPT2 during pregnancy**		
No	35 (191)	(31–40)
Yes	65 (360)	(60–69)
**Delivered from health facility**		
No	43 (234)	(39–48)
Yes	57 (317)	(52–61)
**Exclusively breastfed**		
No	43 (259)	(39–48)
Yes	56 (292)	(52–61)
**Postnatal care (maternal) within 48 h by an appropriate provider**		
No	71 (397)	(65–75)
Yes	29 (154)	(25, 34)

*CI, 95% confidence interval*.

*Design was a cross-sectional survey using lot quality assurance sampling method*.

### Utilization of Maternal Health Services

As shown in Table 1, the proportion of women who had received ANC at least once for their most recent birth in the past 5 months preceding the survey was 69% (95% CI: 65–73). However, the proportion of mothers that had attended the WHO-recommended focused ANC was much lower, at only 29% (95% CI: 25–34). The proportion of women who had delivered in a health facility was 57% (95% CI: 52–61), and only 39% (95% CI: 34–44) reported receiving assistance at childbirth by skilled personnel. Our results also show that only 29% (95% CI: 25–34) of women had PNC within 48 h.

### Predictors of Utilization

#### Single Level Analysis

Education was an important predictor for focused ANC and SBA at delivery, as shown in Table 2. Women who have been to school were 2.17 times and 2.46 times more likely to attend focused ANC and SBA at delivery, respectively.

**Table 2:** **Univariate analysis [odds ratio (OR) with 95% confidence interval (95% CI)] of the predictors of the indicators of the use of maternal health-care services among the poor rural populations of Zambia**.

Factors	Received antenatal care (ANC) at least four times (FANC)			Skilled attendance at delivery			Received postnatal care within 48 h after delivery		
	*n*/*N*	OR (95% CI)	*P*-value	*n*/*N*	OR (95% CI)	*P*-value	*n*/*N*	OR (95% CI)	*P*-value
**Age**									
15–24	57/218	1		91/218	1		62/218	1	
24–40	93/287	1.40 (0.88–2.23)	0.154	102/287	0.71 (0.46–1.11)	0.138	80/287	0.84 (0.52–1.35)	0.468
41–54	11/27	2.13 (0.76–6.05)	0.152	11/27	1.26 (0.46–3.45)	0.649	9/27	1.50 (0.52–4.35)	0.451
**Marital status**									
Married	56/211	1		84/211	1		68/211	1	
Not married	109/340	1.48 (0.92–2.4)	0.101	125/340	1.45 (0.94–2.21)	0.088	86/340	1.22 (0.77–1.91)	0.393
**Ever been to school**									
No	21/110	1		28/110	1		25/110	1	
Yes	143/438	2.17 (1.23–3.83)	0.008	181/438	2.46 (1.42–4.26)	0.001	129/438	1.38 (0.79–2.44)	0.260
**Literacy**									
Cannot read at all	92/351	1		111/351	1		76/351	1	
Only parts	17/53	1.85 (0.90–3.79)	0.093	28/53	2.90 (1.45–5.77)	0.003	20/53	2.07 (1.02–4.22)	0.045
Whole sentence	56/147	1.71 (1.05–2.80)	0.032	70/147	2.21 (1.38–3.53)	0.001	58/147	2.12 (1.27–3.53)	0.004
**Took the HIV test**									
No	16/124	1		13/124			6/124		
Yes	149/427	3.53 (1.91–6.55)	0.000	196/427	5.79 (2.87–11.67)	0.000	148/427	6.88 (2.58–18.38)	0.000
**Received HIV test results**									
No	30/154	1		21/154	1		14/154	1	
Yes	135/397	2.17 (1.3–3.58)	0.003	188/397	4.97 (2.79–8.84)	0.000	140/397	4.48 (2.26–8.86)	0.000
**Received IPT2 during pregnancy**									
No	29/191	1		49/191			42/191		
Yes	136/360	3.59 (2.14–6.00)	0.00	160/360	1.75 (1.11–2.75)	0.015	112/360	1.22 (0.74–1.99)	0.421
**ANC once with a skilled attendance**									
No	**	**	**	172 (342)	1		218 (397)	1	
Yes				184 (209)	5.93 (3.50–10.05)	0.000	137 (154)	5.63 (3.09–10.27)	0.000
**ANC at least four time**									
No	**	**	**	122/386	1		89/386	1	
Yes				87/165	2.41 (1.66–3.50)	0.000	65/165	1.50 (0.95–2.38)	0.081
**Skilled birth attendants**									
No	**	**	**	**	**	**	20/342	1	0.000
Yes							134/209	40.19 (21.45–75.31)	0.000
**Delivery at health facility**									
No	**	**	**	**	**	**	3/234	1	
Yes							151/317	96.81 (27.98–134.96)	0.000
**Community-level factors**									
Type of residence									
Remote rural	137/455	1		153/455	1		115/455	1	
Central rural	28/96	0.93 (0.55–1.60)	0.80	56/96	3.11 (1.89–5.10)	0.000	39/96	2.02 (1.27–3.20)	0.004
**Ratio of health facilities to population in the SA**									
Up to 10,000	92/285	1		106	1		74/285	1	
>10,000	73/266	0.84 (0.55–1.29)	0.426	103	1.15 (0.77–1.71)	0.487	80/266	1.50 (0.96–2.33)	0.075

*Statistical significance at P < 0.05; CI, confidence interval*.

**Variables that could not be regressed against the other variable.

As shown in Table 2, women who could read the whole sentence were 1.71, 2.21, and 2.12 times (*P* < 0.05) more likely to use focused ANC, SBA, and PNC within 48 h, respectively, than women who could not read.

Furthermore, women who received ANC at least once from a skilled personnel (*P* < 0.01) and at least four times (*P* < 0.01) were more likely to have received assisted skilled delivery. Mothers who took an HIV test and those who received the HIV test results (*P* < 0.01) were more likely to be users of SBA and PNC. Most of the predictor variables for SBA were also significantly associated (*P* < 0.01) with use of PNC within 48 h of delivery by a skilled provider (see Table 2). In addition, women who had SBA at the health facility were more likely (*P* < 0.01) to use PNC within 48 h after delivery. Under the community-level predictors, our analysis shows that women who live in central rural areas were more likely to use SBA and PNC within 48 h after birth than those living in remote rural areas (*P* < 0.01). For instance, women living in central rural areas were 3.11 times more likely to receive SBA than women living in remote rural communities. However, our analysis did not find substantial association between the ratios of PHC to community population with the use of all the three indicators of maternal health care services.

### Multilevel Analysis

For the initial multilevel analysis, we started with an empty random two-level intercept model to test the null hypothesis that there is no variation in the effects of maternal health service utilization between community clusters, the results of which show considerable variation across the communities, particularly for SBA. Based on the VPC values in the empty model shown in Table 3, 9% of the total variance in the use of focused ANC, 27% variance in the use of SBA, and 22% variance in the use of PNC within 48 h were attributable to the unobserved differences across community clusters. Furthermore, the results show significantly reduced odds for utilizing focused ANC, SBA, and PNC within 48 h by 60% (OR 0.40; 95% CI: 0.30–0.53), 47% (OR 0.53; 95% CI: 0.34–0.84), and 67% (OR 0.33; 95% CI: 0.22–0.49), respectively, across the communities. See Table 3.

**Table 3:** **Parameter coefficients for the multilevel model for various indicators of the use of maternal health services—empty model without covariates**.

	Antenatal care	Skilled attendance at delivery	Postnatal care
**Level 2 variance—random effects**			
Community (SA) odds ratio (CI)	0.40 (0.30–0.53)	0.53 (0.34–0.84)	0.33 (0.22–0.49)
Community (SA) VPC (%)	9	27	22

*CI, confidence interval; VPC, variance partition coefficients*.

After the empty two-level intercept modeling with only random effects, a full multilevel model was run to assess predictors of the three indicators of maternal health-care services. The model included fixed effects at individual and community levels, and the findings are presented in Table 4.

**Table 4:** **Results of the multilevel analysis of the factors related to the use of maternal health services among the poor rural populations of Zambia**.

Variables	Adjusted odds ratios (CI)			
	Characteristics	Antenatal care at least four times (FANC)	Skilled attendance at delivery	Postnatal care
**Fixed effects**				
Individual variables				
Age	15–24	1	1	1
	24–40	1.78 (1.13–2.81)*	0.81 (0.50–1.31)	1.42 (0.77–2.61)
	41–54	4.04 (1.52–10.78)*	1.69 (0.58–4.94)	2.09 (0.53–8.25)
Marital status	Not married	1	1	1
	Married	1.43 (0.84–2.44)	0.97 (0.54–1.77)	0.69 (0.35–1.39)
Ever attended school	No	1	1	1
	Yes	3.47 (0.48–24.98)	2.28 (0.27–19.44)	0.13 (0.02–1.84)
Literacy	Cannot read at all	1	1	1
	Only parts	1.12 (0.53–3.86)	2.73 (1.23–6.18)**	1.34 (0.52–3.45)
	Whole sentence	1.21 (0.65–2.38)	1.29 (0.68–2.44)	1.63 (0.72–3.68)
Took HIV test	No	1	1	1
	Yes	5.73 (2.09–15.77)**	2.21 (1.70–4.93)*	6.18 (1.20–28.16)*
Received HIV test results	No	1	1	1
	Yes	0.38 (0.16–0.91)*	1.98 (0.75–5.27)	0.65 (0.19–2.20)
Received intermittent preventive treatment 2	No	1	1	1
	Yes	3.62 (2.10–6.21)***	1.56 (0.93–2.64)	0.69 (0.34–1.395)
ANC1 with skilled personnel	No		1	1
	Yes		4.48 (2.38–8.43)***	2.42 (1.40–5.62)*
ÀNC4	No		1	1
	Yes		1.05 (0.62–1.77)	0.87 (0.44–1.72)
Delivery at a health facility	No			1
	Yes			17.42 (4.67–54.95)***
Skilled birth attendance	No			1
	Yes			9.48 (4.42–20.34)***
**Community (SA) level variables**				
Type of residence	Remote rural	1	1	1
	Central rural	0.73 (0.34–1.54)	2.57 (1.07–6.50)*	0.88 (0.33–2.36)
Ratio of health facilities to population in the SA	Up to 10,000	1	1	
	>10,000	1.02 (0.57–1.81)	0.93 (0.45–1.93)	1.50 (0.69–3.25)
**Random effects**				
Community (SA) random variance (SE)		0.245 (0.16)	0.544 (0.28)	0.428 (0.29)
Community (SA) VPC (%)		7	14	12

*SA, supervisory area; VPC, variance partition coefficients*.

*P < 0.05.

**P < 0.01.

***P < 0.001.

### Antenatal Care

As shown in Table 4, compared to younger women, older women were four times (OR 4.04; 95% CI: 1.52–10.78) more likely to attend ANC at least four times.

Furthermore, the multilevel analysis indicates a positive association (*P* < 0.01) between focused ANC and taking an HIV test, receiving HIV test results, and receiving IPTp2 for malaria at least twice. Although statistically insignificant, the odds of reporting use of ANC by women who had ever attended school was 3.47 times higher (OR 3.47; 95% CI: 0.48–24.98).

At community level, the type of residence (that is women in centralized rural areas) shows a negative trend toward utilization of focused ANC, though results were statistically non-significant. The ratio of PHCs to the population in the community cluster above 10,000 people did not predict use of ANC. Our results show a marginal 2% decrease, from 9 to 7%, of the VPC for use of ANC, indicating a very minimal decrease in the clustering of ANC utilization even after controlling for individual- and community-level variables.

### Skilled Birth Attendance

The significant individual-level variables that predict SBA included; literacy, ANC attendance, and having received HIV test results, as shown in Table 4. Women who could read some parts of a sentence were positively associated with SBA (OR 2.73; 95% CI: 1.23–6.18). Having received at least one ANC by a skilled provider in the recent pregnancies had a positive influence (OR 4.48; 95% CI, 2.38–8.43) on SBA. Women who had received HIV test results had a 3.80 likelihood of SBA. As shown in Table 4, compared to women residing in remote rural areas, women in central rural areas were 2.72 times (OR 2.57; 95% CI: 1.37–6.82) more likely to have a delivery by a skilled attendant. The VPC in the use of skilled delivery reduced from 27% in the empty model to 14%, indicating continued clustering of utilization of SBA even after controlling for individual- and community-level factors.

### Postnatal Care

Use of at least one ANC provided by skilled personnel, having taken an HIV test during pregnancy, SBA, and delivering at a health facility were the only predictor variables that were significantly associated with utilization of PNC by women within 48 h of delivery. As shown in Table 4, the odds of using PNC within 48 h of delivery by an appropriate provider was 2.42 times (OR 2.42; 95% CI: 1.04–5.62) among women who received at least one ANC by a skilled personnel and 9.48 times (OR 9.48; 95% CI: 4.42–20.33) among women that had skilled assistance at delivery.

Likewise, a positive association was found between the use of PNC and delivering at a health facility. Our analysis did not find a significant influence in the use of PNC by the type of residence or the ratio of PHCs to population in the community cluster.

## Discussion

In this study, we sought to investigate utilization patterns and determinants of the three indicators of continuity of care for maternal health-care services, ANC, SBA, and PNC, among the most remote, rural, and poor communities of Zambia, and five key lessons were observed. First, the remote rural mothers living in poor communities had much lower utilization rates of maternal health-care services compared to the national average for rural Zambia, they were therefore worse off given their marginalized location. Second, both focused ANC and SBA were predicted by an HIV test when HIV test results are also provided to mothers, indicating the importance of HIV testing as a driver of service utilization. Third, a single ANC provided by a skilled health worker was a predictor of both SBA and PNC within 48 h, a potential bridge for the lower rates of focused ANC in these communities. Fourth, women in remote rural areas were found to be at a disadvantage, and less likely to utilize SBA when compared to women in centralized rural areas. Finally, PNC within 48 h was predicted by both SBA and delivery at a health facility, reiterating the importance of institutional delivery. However, less than 40% of women in this study had a SBA, suggesting probable missed opportunities in PNC for the 60% of women who did not have a SBA.

Overall, utilization of the three indicators of maternal health services in these rural and remote populations was very low, with only a third of women receiving focused ANC and PNC within 48 h and less than half receiving SBA. These coverage levels are much lower than the national estimates for rural areas in Zambia (11) and other sub-Saharan African countries (2, 10, 12, 14, 28). For instance, compared to the ZDHS national estimates of 55.2% of women in rural areas attend at least four ANC visits 29% of women in remote areas attended ANC at least four times.

Meanwhile, 39% of women in these remote communities had a skilled attendant at delivery compared to the ZDHS national estimates of 52% of women delivered by a skilled provider in rural areas.

At individual level, attendance of ANC at least four times was found to be positively influenced by the age of the woman, having taken an HIV test, having received results for an HIV test, and having received prevention for malaria. It was not surprising that older women were more likely to use ANC services given that previous studies have reported a positive association between the age of the woman and use of ANC in similar contexts (12, 28–30). Older women are also more knowledgeable about health-care services (30), have greater decision-making power than younger women, and are thus benefiting from using such services, hence the higher likelihood of utilization (31). In addition, older age as a predictor of utilization of ANC services could also be related to greater likelihood of having a previous birth and interaction with services. However, some studies show a lack of association between maternal age and health service utilization (32).

Findings regarding a positive association between the number of ANC visits attended and having tested for HIV are consistent with other studies in the region (33, 34). Routine HIV testing during ANC visits seems to provide a condition for attending ANC, and therefore, women who attend ANC are more likely to be tested (35).

Consistent with prior research in rural Kenya (36) and elsewhere (10, 30, 37, 38), this study found literacy status of women as an influential predictor for the use of SBA. Women that are able to read are more likely to have better access to information on health matters. It is also likely that literate women have more confidence, are capable in making decisions about their health (28, 36), have better socioeconomic status, and are better placed to overcome cultural barriers to maternal health-care use (16, 39).

Some researchers, however, question the independent association of literacy on MNH services utilization. They argue that other factors such as husband’s educational level and socioeconomic environment interact to dilute the association (40, 41).

Most notably, our study found that receiving at least one ANC from a skilled provider had a noteworthy effect on the use of both SBA and PNC within 48 h of child birth, a finding supported by only one other study in Ethiopia (13). The positive contribution of at least one ANC visit by a skilled provider on use of SBA and PNC, observed in rural and remote areas of Zambia where distance is a challenge to accessing services (42), may have implications to policy and practice. These findings are contrary to what most studies have reported that women who attend less than the recommended four ANC visits are less likely to receive SBA (10, 31).

As observed in previous studies (43, 44), our study confirms the significant association of previous use of maternal health services on subsequent services. The predictor SBA in this study demonstrates that women tend to use PNC services if they were attended by skilled personnel at delivery. One of the expected effects of giving birth with skilled attendants is that it reinforces the use of PNC services. Worku et al. (13) argue that women who tend to use maternal health-care services build confidence in skilled providers and their care. Moreover, based on the effect of use of ANC on SBA and PNC found in this study and elsewhere (44), it seems right to consider ANC visits as a feasible entry point for subsequent utilization of the indicators of maternal health services.

Consistent with other studies (12, 13), finding that women who live in central rural areas were more likely to attend an assisted delivery, is an indicator of inequity for those mothers located on the margins of society, and socially excluded from maternal health care services. Some studies have also shown that within the poor rural areas, the patterns of maternal health care service utilization vary widely (3, 12, 45).

This could be a reflection of varying contextual and geo-social differences that span across these communities even if they may all be grouped as poor and vulnerable populations. Therefore, context matters, and an understanding of the context of the variations in the use of maternal health services based on place of residence is required to design interventions that address inequalities, as argued by Say and Raine (46).

### Limitations

This study may have suffered from some limitations that could have affected our findings. Selection bias could have occurred as a result of the cross-sectional design used for this study, and the use of LQAS, which has been designed to measure group variables rather than individual variables. Inclusion of only women with surviving infants could have also led to selection bias. Also, measurement biases could have occurred due to limited variables, missing others that could have explained other associations, such as economic status and distance to the health facility. The data analyzed was self-reported and therefore subject to social desirability and recall bias, though women were interviewed within 5 months of childbirth. However, although we think that these biases were present, we do not think they are important in explaining the findings. First, pregnancy and childbirth are revered in these rural communities, such that women would not easily forget their experiences. Furthermore, we used multilevel modeling to control for the effects of between and within group variability, and estimated the effects of group (cluster) level characteristics on outcomes. Notably, using LQAS-based data to measure individual effects in this study, considering the original aim of such methodology, is a novel and demand driven approach. The use of LQAS as a rapid methodology for monitoring programs is a feasible and quick measure for obtaining data on a large scale, and as such, could be used to rapidly inform interventions and policies without waiting for the slow expensive demographic and health surveys currently being employed. For these reasons, we think that these results can be extrapolated to similar communities in Zambia, not withstanding differential contextual contrasts that may exist. In view of such variations, generalizing these results in other low income settings must be done with caution.

## Conclusion

In this study, we have found that utilization of key indicators of maternal health services among the remote and poorest rural populations of Zambia is very low and much lower than the national estimates. Only a third of mothers achieved the WHO-recommended focused ANC, with a third of mothers not having been to ANC at all. Women were more likely to attend focused ANC when they were offered an HIV test and given results for HIV tests, indicating the importance of HIV testing as a driver of service utilization. Importantly, we have found that receiving ANC once from a skilled attendant is a significant predictor of both SBA and PNC within 48 h, by women in these remote and poorest populations of Zambia, a potential bridge for the lower rates of focused ANC in these communities. Based on these results, contact with skilled health-care providers seems very important for use of services in these rural remote communities. Our finding of remote rural communities being at a disadvantage, and less likely to receive SBA when compared with centralized rural areas, suggests that disparities in the use of MH services do exist within rural populations.

Therefore, we recommend that remote and poor women, who are unable to frequently attend ANC services, may benefit from SBA and PNC by attending at least one ANC visit offered by a skilled provider. We also recommend further research to understand the perspectives of women toward focused ANC and explore the barriers to utilization of focused ANC in the remote and poorest rural populations.

## Author Contributions

CJ, CM, and MM conceptualized the study. CJ, CM, NL, and SM participated in data collection and cleaning. CJ, CM, NL, and SM participated in designing the study. CJ, MM, and CM participated in data analysis. CJ prepared the first draft of the manuscript. MM and CM provided scientific advices on data analysis and throughout the preparation of the manuscript. All the authors worked on revising various drafts of the manuscript. All the authors read and approved the final draft.

## Conflict of Interest Statement

The authors declare that the research was conducted in the absence of any commercial or financial relationships that could be construed as a potential conflict of interest. The reviewers HT and LK and handling Editor declared their shared affiliation, and the handling Editor states that the process nevertheless met the standards of a fair and objective review.

## Abbreviations

ANC, antenatal care; SBA, skilled birth attendance; PNC, postnatal care; IPTp, intermittent preventive treatment; HIV, human immunodeficiency syndrome; LQAS, lot quality assurance sampling; PHC, primary health care; WHO, World Health Organization.
